# DE-Sync: A Doppler-Enhanced Time Synchronization for Mobile Underwater Sensor Networks

**DOI:** 10.3390/s18061710

**Published:** 2018-05-25

**Authors:** Feng Zhou, Qi Wang, DongHu Nie, Gang Qiao

**Affiliations:** 1 Acoustic Science and Technology Laboratory, Harbin Engineering University, Harbin 150001, China; zhoufeng@hrbeu.edu.cn (F.Z.); tony.wangqi@hrbeu.edu.cn (Q.W.); qiaogang@hrbeu.edu.cn (G.Q.); 2 Key Laboratory of Marine Information Acquisition and Security (Harbin Engineering University), Ministry of Industry and Information Technology, Harbin 150001, China; 3 College of Underwater Acoustic Engineering, Harbin Engineering University, Harbin 150001, China

**Keywords:** doppler, sensor node, time synchronization, mobility, propagation delay

## Abstract

Time synchronization is the foundation of cooperative work among nodes of underwater sensor networks; it takes a critical role in the research and application of underwater sensor networks. Although numerous time synchronization protocols have been proposed for terrestrial wireless sensor networks, they cannot be directly applied to underwater sensor networks. This is because most of them typically assume that the propagation delay among sensor nodes is negligible, which is not the case in underwater sensor networks. Time synchronization is mainly affected by a long propagation delay among sensor nodes due to the low propagation speed of acoustic signals. Furthermore, sensor nodes in underwater tend to experience some degree of mobility due to wind or ocean current, or some other nodes are on self-propelled vehicles, such as autonomous underwater vehicles (AUVs). In this paper, we propose a Doppler-enhanced time synchronization scheme for mobile underwater sensor networks, called DE-Sync. Our new scheme considers the effect of the clock skew during the process of estimating the Doppler scale factor and directly substitutes the Doppler scale factor into linear regression to achieve the estimation of the clock skew and offset. Simulation results show that DE-Sync outperforms existing time synchronization protocols in both accuracy and energy efficiency.

## 1. Introduction

In recent years, underwater sensor networks have played an important role in coastal surveillance, environmental monitoring, undersea exploration, disaster prevention, mine exploration, and other fields. Considerable attention has been paid to the research and application of underwater sensor networks. However, because of the characteristics of underwater acoustic channels, the research and application of underwater sensor network communication has suffered from many challenges [1,2]. The time synchronization of underwater sensor networks, as one of the supporting technologies, is also very different from the time synchronization of terrestrial wireless sensor networks.

Time among nodes is inconsistent due to the difference of angular frequency of the crystal oscillator and the difference of boot time of the distributed systems, in which nodes go out of synchronization as time elapses. Most applications in networks require that all sensor nodes have a common notion of time. Depending on the time synchronization level required, these applications can be classified into three categories [3]. Some applications only require the order of events that occur, while other functionalities require the time interval of each of the occurrences. There are also some applications that require an absolute time at which each event occurs, and the implementation of these applications depends on a precisely synchronized time among network nodes. For example, the time-sensitive data with time stamping information from multiple sensor nodes are then aggregated to a cluster head node, and other applications such as node tracking and location require precise timing. In addition, not only would the application layer require time synchronization; other layers, such as the medium access control (MAC) layer, also have considerable functionalities that benefit from time synchronization.

Numerous time synchronization protocols for terrestrial wireless sensor networks have been proposed in various publications, such as the fine-grained network time synchronization using reference broadcasts (RBS) [4], the timing-sync protocol for sensor networks (TPSN) [5], the flooding time synchronization protocol (FSTP) [6] and others [7,8,9,10]. However, these protocols cannot be directly applied to underwater sensor networks. This is because they assume that the propagation delay among nodes is negligible. However, in underwater acoustic communication, with a propagation speed (roughly 1500 m/s in water) of nearly five orders of magnitude lower than terrestrial radio freqency (RF), assumptions about rapid communication are incorrect. Meanwhile, due to the mobility of the sensor nodes that are not propelled or self-propelled, the propagation delay among nodes in water is a dynamic variation, which should be considered in the new approach. In addition, the terrestrial synchronization algorithms typically allow nodes to perform frequent re-synchronization and are not highly dependent on available bandwidth and energy constraints. In contrast, underwater acoustic communication systems may have a bandwidth of only a few hundred kilohertz, while bandwidth would drop to a few kilohertz if the targeted communication range reaches several tens of kilometers [1]. So the bandwidth of the system restricts time synchronization overhead that is not sufficient for frequent re-synchronization. Furthermore, nodes restricted by energy constraints and recharging limitations should avoid frequent re-synchronization.

Time synchronization solutions for underwater sensor networks aim to solve the challenges, including the long and dynamic propagation delay, and especially the dynamic propagation delay caused by the mobility among nodes [11,12]. This is an important issue on which recent synchronization schemes focus. In this paper, through analyzing the existing protocols, we propose a Doppler-enhanced time synchronization scheme for mobile underwater sensor networks, called DE-Sync. Our new scheme is a novel synchronization solution for the context of the dynamic propagation delay.

Different from the existing time synchronization protocols based on the Doppler effect, DE-Sync has two main distinguishing attributes. First of all, DE-Sync considers the effect of the clock skew during the process of estimating the Doppler scaling factor, which compensates for the estimation error introduced by the clock skew and increases the accuracy of estimation in the Doppler scale that affects the accuracy of time synchronization. Secondly, the Doppler scaling factor, instead of the relative moving speed between nodes, is contained in the exchange messages, which reduces the quantity of division computations. Because the acoustic velocity in water is a function of depth, temperature and salinity, our new approach does not directly involve the calculation of acoustic velocity, which reduces the error of estimating the clock skew and offset during the process of linear regression, and improves the accuracy of time synchronization. In our comparison of simulation results, the performance of DE-Sync protocol is better than that of existing time synchronization protocols for mobile underwater sensor networks, in both accuracy and energy efficiency.

DE-Sync proposed in this paper deals with time synchronization based on Doppler scale estimation. For underwater acoustic communication, Doppler scale estimation is typically a critical research topic. There are two popular methods for Doppler scale estimation [13]. One method is to send Doppler insensitive signals for Doppler scale estimation [14,15,16,17]. Another method is to send a Doppler sensitive waveform for Doppler scale estimation [18,19,20,21]. Both of the two methods have a common application limitation, which is not ideal for real time processing of the estimation algorithm. DE-Sync adopts a combined Doppler scale estimation scheme that involves two steps to enhance the accuracy of the Doppler scale factor acquired in real time [22,23,24]. In the first step, one linear frequency modulation (LFM) and two identical short orthogonal frequency-division multiplexing (OFDM) symbols preceded by a cyclic prefix (CP), are used as a preamble for initial Doppler scale estimation. In the second step, fine Doppler scale estimations can be achieved based on the CP of each CP-OFDM block.

The remainder of this paper is organized as follows. In Section 2, we review the previous works of the existing time synchronization protocols for underwater sensor networks. We then provide a description of DE-Sync in Section 3. Simulation results are shown in Section 4. Finally, we give our conclusions in Section 5.

## 2. Related Work

The design of time synchronization protocol for underwater sensor network needs to consider two main factors, one is the long propagation delay during communication between nodes, and the other is the relative mobility between nodes. At present, a few time synchronization protocols for underwater sensor networks have been proposed, such as the time synchronization for high latency acoustic networks (TSHL) [25], the time synchronization protocol for underwater mobile networks (MU-Sync) [26], the efficient time synchronization for mobile underwater sensor networks (Mobi-Sync) [27], the Doppler-based time synchronization for mobile underwater sensor networks (D-Sync) [28], and the time synchronization scheme for mobile underwater sensor networks (TSMU) [29]. However, these protocols have their own application scenarios and they all make different assumptions.

TSHL was the first time a synchronization algorithm was designed for high latency networks, networks in which the algorithm is organized in two phases. TSHL assumes the constant propagation delay among sensor nodes and, essentially, they assume a static network; however, nodes typically move with the ocean current (around 0.83–1.67 m/s) [30], especially in the case of self-propelled vehicles which move faster, such as autonomous underwater vehicles (AUVs); therefore, the protocol cannot handle mobile scenarios.

MU-Sync takes account of time variability in the propagation delay due to the relative motion of nodes using a two-phase operation, namely the skew and offset acquisition phase, and the synchronization phase. MU-Sync assumes that the one-way propagation delay can be calculated as half of the round trip time, which significantly deteriorates the performance of MU-Sync when the nodes move fast, especially when the unsynchronized nodes respond to the cluster head for a long period of time.

Mobi-Sync introduces spatial correlation of the nodes’ velocities to estimate the varying time propagation delay. In the protocol, all nodes are classified into three categories: surface nodes, super nodes and ordinary nodes. Surface nodes are equipped with a global positioning system (GPS) to obtain the global time reference, while super nodes can communicate directly with surface nodes to achieve time synchronization. However, Mobi-Sync applies only to certain special scenarios, which is because they assume that the exact correlation among neighboring nodes is known, which is difficultly obtained for many actual networks such as those with a long distance between nodes or on self-propelled vehicles.

D-Sync leverages the Doppler shift caused by the relative motion of nodes in underwater environments. D-Sync does not consider the effect of the skew during the process of estimating the Doppler scaling factor, which reduces the accuracy of the Doppler shift estimation and affects the accuracy of time synchronization. Therefore, as the initial skew increases, the accuracy of time synchronization deteriorates.

TSMU utilizes the Doppler effects caused by the relative motion between nodes during the process of time synchronization. Meanwhile, they consider the effect of the skew during the process of estimating the Doppler scaling factor from the physical layer. Furthermore, the Kalman filter and calibration process are exploited during the process of time synchronization. However, due to the velocity of sound in water as a function of depth, temperature and salinity, the estimation of sound velocity is directly applied into the computation during the process of synchronization, causing extra errors. In addition, there are three parameters to be adjusted, initial skew, etc., which affect the accuracy of synchronization and efficiency.

The closest work to our proposed solution is D-Sync, and there are two key differences between DE-Sync and D-Sync: one is that DE-Sync considers the effect of the clock skew during the process of estimating the Doppler scaling factor, and the other is that DE-Sync uses the Doppler scaling factor instead of the relative moving speed among nodes when performing linear regression. Therefore, a performance comparison between DE-Sync and D-Sync is given in Section 4. 

## 3. Protocol Description

### 3.1. Overview of the DE-Sync

The clock timer inside each node uses a crystal oscillator operating at a certain angular frequency, which determines the rate at which the clock runs [3]. Typically, the clock inside each node has an intrinsic drift in angular frequency due to the manufacturing process. The numerous synchronization protocols use a common time model that DE-Sync also follows to estimate the skew and offset. The local time of any node is relevant to the reference time by Equation (1).

(1) T=αt+β

where *T* denotes the local time of nodes at time *t*, *t* is the reference time, i.e., beacon time. *α* and *β* are the relative clock skew and the offset, respectively. Moreover, *α* is related to the angular frequency of the crystal oscillator and *β* is affected by the starting time of the system.

The time synchronization procedure of DE-Sync consists of three phases: data collection, linear regression and calibration. In the data collection phase, the unsynchronized node collects the time stamps from synchronization messages as well as their Doppler scale factors, with two-way synchronization message exchanges. In the linear regression phase, the unsynchronized node performs the first linear regression with LS (least square estimation) to estimate the clock skew and offset. In the calibration phase, the unsynchronized node performs the calibration process by updating the initial skew, and re-performs the synchronization process to obtain the final clock skew and offset. All parameters are listed in Table 1.

### 3.2. Impact of the Clock Skew

Let us assume that beacon node *A* is used as a reference node which has an ideal clock and node *B* is an unsynchronized node which has a drifting time—a skew with respect to an ideal clock. Let node *B* be synchronized with node *A*. The signal received from the baseband, going from node *A* to node *B*, is given by Equation (2), which can be formulated as the summation of signals arriving along multiple physical paths [29,31].

(2) $$y_{AB}\left(n\right)=\overset{N_{p}}{\underset{p=1}{\sum }}A_{p}x_{AB}\left(\left(1+a_{AB}\right)n-\tau_{p}\right)$$

In this equation, *N_p_* stands for the path number, *a_AB_* denotes the measured Doppler scale factor, and *A_p_* and *τ_p_* denote amplitude and delay of the *p*th path, respectively. Based on Equation (1), due to a drift of the skew between the clocks of nodes, when the time in node *A* is expressed as $t[n]=nT_{s}$, the basis time in node *B* can be written as $t[n]=\alpha \left(nT_{s}\right)$. *a_AB_* depends on both relative motion speed and clock skew difference among nodes, and it is expressed as:

(3) $$1\;+\;a_{AB}=\alpha \left(1+a_{m}\right)$$

where *a_m_* denotes the Doppler scale factor induced by the node mobility. Similarly, upon receiving the synchronization message sent from node *B*, the basis time in node *A* can be written as t[n]=(nTs)α, the Doppler scale factor measured by node *A*, namely *a_BA_*, is expressed as:

(4) $$1\;+\;a_{BA}=\frac{\left(1\;+\;a_{m}\right)}{\alpha }$$

So, based on the measurement of the Doppler scale factor, *a_m_* is expressed as:

(5) am={((1 + aAB)α)−1A→Bα(1 + aBA)−1B→A

As a result, since the clock skew *α* in the initial phase is unknown, *α* is assigned with an initial value “1”. After the first linear regression, *a_m_* can be calibrated based on the estimation of *α*. Moreover, *a_m_* is used to re-perform the synchronization process to obtain the final clock skew and offset.

### 3.3. Details of the DE-Sync

**Figure 1:**
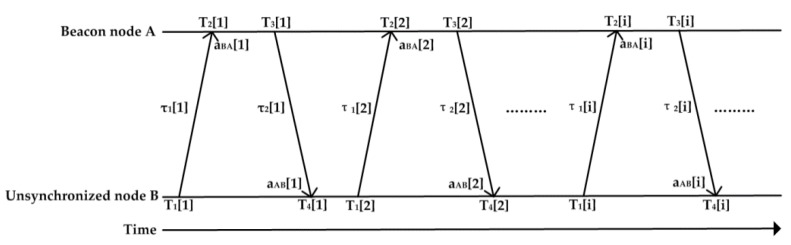
DE-Sync Message Exchange Scheme.

In DE-Sync, the synchronization process is initiated by the unsynchronized node. When the unsynchronized node *B* moves to the coverage area of the underwater sensor networks, it sends a synchronization request to beacon *A*. Meanwhile, node *B* records the sending time-stamp *T*_1_ obtained from the MAC layer right before the message departs. Upon receiving the synchronization request, beacon *A* marks its local time as the receiving time-stamp *T*_2_ and records the Doppler scale factor *a_AB_* obtained from the physical layer. Then, beacon *A* needs to back off a random time, namely *T_backoff_*, to avoid message collisions before it transmits a response message back to node *B* at time *T*_3_, which contains *T*_2_, *T*_3_ and *a_BA_*. As before, when receiving the synchronization response message, node *B* records its receiving time and the Doppler scale factor *a_AB_*. After a few rounds of message exchanges, node *B* will collect a set of time-stamps consisting of *T*_1_, *T*_2_, *T*_3_ and *T*_4_, and the measured Doppler scale factor consisting of *a_BA_* and *a_AB_*. Figure 1 shows the message exchange process between node *A* and node *B*.

Now, we will derive a set of equations used by DE-Sync to synchronize nodes.

As given by Equation (1), the following set of equations of the node’s local time can hence be derived:

$$T_{1}\;=\;\alpha t_{1}\;+\;\beta \;T_{2}{\;=\;t}_{2}\;T_{3}{\;=\;t}_{3}\;T_{4}\;=\;\alpha t_{4}\;+\;\beta$$

*t*_1_, *t*_2_, *t*_3_ and *t*_4_ denote the reference time, and they derive the following relationship:

$$t_{2}{\;=\;t}_{1}\;+\;\tau_{1}\;t_{4}{\;=\;t}_{3}\;+\;\tau_{2}$$

Hence, the local time of node *A* and node *B* can be expressed as:

(6) $$\begin{array}{l} T_{1}=\alpha \left(T_{2}-\tau_{1}\right)+\beta \\ T_{4}=\alpha \left(T_{3}\;+\;\tau_{2}\right)+\beta \end{array}$$

Considering sensor node mobility, propagation delay *τ*_1_ is not essentially equivalent to *τ*_2_, and based on Equation (6), we get:

(7) τ1+τ2=((T4−T1)α)+T2−T3τ2−τ1=Δd/cΔd=∫t2t4vAB(t) dt

Let *v_m_* be the average of relative speed among nodes from *t*_2_ to *t*_4_, hence $\Delta d{=v}_{m}\left(t_{4}{-t}_{2}\right)$. Combining with (6), we get:

(8) $$\tau_{2}-\tau_{1}=\frac{v_{m}}{c}\times \;\left(\left(\frac{T_{4}-\beta }{\alpha }\right)-T_{2}\right)$$

If we let $\theta =\frac{v_{m}}{c}$, and combine Equations (7) and (8), then *τ*_1_ and *τ*_2_ are solved as:

(9) $$\begin{array}{l} \tau_{1}=\frac{T_{4}\left(1-\theta \right)-T_{1}+\theta \beta }{2\alpha }+\frac{T_{2}\left(1+\theta \right)}{2}-\frac{T_{3}}{2}\\ \tau_{2}=\frac{T_{4}\left(1+\theta \right)-T_{1}-\theta \beta }{2\alpha }+\frac{T_{2}\left(1-\theta \right)}{2}-\frac{T_{3}}{2} \end{array}$$

*τ*_1_ and *τ*_2_ are substituted into (6), which give us the following equation:

(10) $$T_{1}+T_{4}\left(1-\theta \right)=\alpha \left(T_{2}\left(1-\theta \right)+T_{3}\right)+\beta \left(2-\theta \right)$$

Node *A* at *t*_2_ measures the Doppler scale factor expressed as aBA=vBAc, and node *B* at *t*_4_ measures the Doppler scale factor expressed as aAB=vABc.

Let the average of *v_AB_* and *v_BA_*, namely interpolation, replace the average of relative speed among nodes. Hence *v_m_* = 0.5 × (*v_AB_* + *v_BA_*) + *ε_v_*, i.e., *v_m_* = 0.5 × *c* × (*a_AB_* + *a_BA_*) + *ε_v_*, where *ε_v_* denotes error due to random noise and the interpolation error. We get:

(11) $$\theta =0.5\times \left(a_{AB}+a_{BA}\right)+\frac{\varepsilon_{v}}{c},$$

and if *θ* is substituted into (10), we get:

(12) $$2T_{1}+T_{4}\left(2-\left(a_{AB}+a_{BA}\right)\right)=\alpha \left(T_{2}\left(2-\left(a_{AB}+a_{BA}\right)\right)+2T_{3}\right)+\beta \left(4-\left(a_{AB}+a_{BA}\right)\right)+\varepsilon$$

Based on Equation (12), we perform linear regression to estimate the skew and offset, which is as follows:

(13) $$\begin{array}{l} \hat{\Lambda }={\left[\hat{\alpha },\hat{\beta }\right]}^{T}={\left(H^{T}H\right)}^{-1}H^{T}Y\\ Y\left[i\right]=2T_{1}{}^{i}+T_{4}{}^{i}\left(2-\left(a_{AB}{}^{i}+a_{BA}{}^{i}\right)\right)\\ H\left[i,1\right]=2T_{3}{}^{i}+T_{2}{}^{i}\left(2-\left(a_{AB}{}^{i}+a_{BA}{}^{i}\right)\right)\\ H\left[i,2\right]=\left(4-\left(a_{AB}{}^{i}+a_{BA}{}^{i}\right)\right) \end{array}$$

As given by (13), after *N* rounds of message exchanges, *Y* is a *N* × 1 vector and *H* is a matrix with *N* × 2 entries. The skew and offset can be estimated by the above equation.

As described earlier, we have discussed the impact of clock skew for the Doppler shift factor, and the skew is assigned with an initial value “1”. However, the skew of the unsynchronized node cannot be 1. In the calibration phase, the initial skew is updated with the estimated skew obtained by using the Equation (13). The unsynchronized node carries out the calibration process to correct the measured Doppler scale factor and re-calculate the clock skew and offset. The calibration process will run iteratively until it reaches the final criterion, which is either when the number of runs reach the maximum iteration (for example: 2, according to the later simulation result) or the difference between estimated skews of two runs is less than the set threshold (for example: 50 part per million (ppm)).

### 3.4. Error Analysis

In DE-Sync, there are two main error sources: the error *ε_noise_* due to random noise in Doppler measurements and the error *ε_interp_* due to the fact that Doppler measurements are not available continuously. In each round of message exchange, the average of the two measured Doppler scale factors, obtained at the end of each message transmission, is used instead of continuous measurements, which lead to the interpolation error. In Equation (11), *ε_v_* is expressed as:

(14) $$\varepsilon_{v}=\varepsilon_{noise}+\varepsilon_{interp}$$

In Equation (12), *ε* is expressed as:

(15) $$\varepsilon =\frac{\varepsilon_{v}}{c}\times \left(T_{4}-\alpha T_{2}-\beta \right)$$

Combining with (6), where *T*_4_ is substituted into (14), we get:

(16) $$\varepsilon =\frac{\varepsilon_{v}}{c}\times \alpha \times \left(T_{3}-T_{2}+\tau_{2}\right)$$

Combining with Equation (14), where *ε_v_* is substituted into (15), we get:

(17) $$\varepsilon =\left(\varepsilon_{noise}+\varepsilon_{interp}\right)\times \frac{\alpha \left(T_{backoff}+\tau_{2}\right)}{c}$$

*T_backoff_* = *T*_3_ − *T*_2_. When considering (17), and the parameters affecting the estimation of error, *ε_interp_* is a main factor. Referring to Equation (7), we get:

(18) $$\varepsilon_{interp}=\frac{1}{t_{4}-t_{2}}\times \int_{t_{2}}^{t_{4}}v_{AB}(t)\;dt-0.5\times \left(v_{AB}+v_{BA}\right)$$

Following the derivation in [28], $\varepsilon_{interp}\leq \frac{\left(t_{4}-t_{2}\right)\times \left(A_{max}{}^{2}-A_{avg}{}^{2}\right)}{4A_{max}}$, *A_max_* and *A_avg_* denote the maximum relative acceleration and average relative acceleration, respectively. Considering equation (18), there is an indication that estimation error in DE-Sync mainly occurs from random noise, i.e., *ε_noise_*, if nodes move at a constant speed or uniform acceleration. *ε_noise_* is a random variable which is subject to Gaussian distribution [20].

## 4. Performance Evaluation

### 4.1. Simulation Setup

First, we compared the performance of DE-Sync and D-Sync because D-Sync is closest to our new scheme; simulation results are obtained with the change of various parameters for a pair of nodes. Furthermore, the post-synchronization error and energy efficiency of the three schemes, namely, DE-Sync, D-Sync and MU-Sync, are compared at the end of this section. In the simulations, the sensor nodes are allowed to move randomly within an area of 1000 m by 1000 m region. The simulation signal exchange scheme is shown in Figure 1. Unless otherwise specified, we use the parameters summarized in Table 2 for our simulations. Without loss of generality, each data point is obtained as an average of the 1000 runs.

Note that all simulation results show the synchronization error 2 h after a node is synchronized.

### 4.2. Simulation Results and Analysis

#### 4.2.1. Number of Calibration

**Figure 2:**
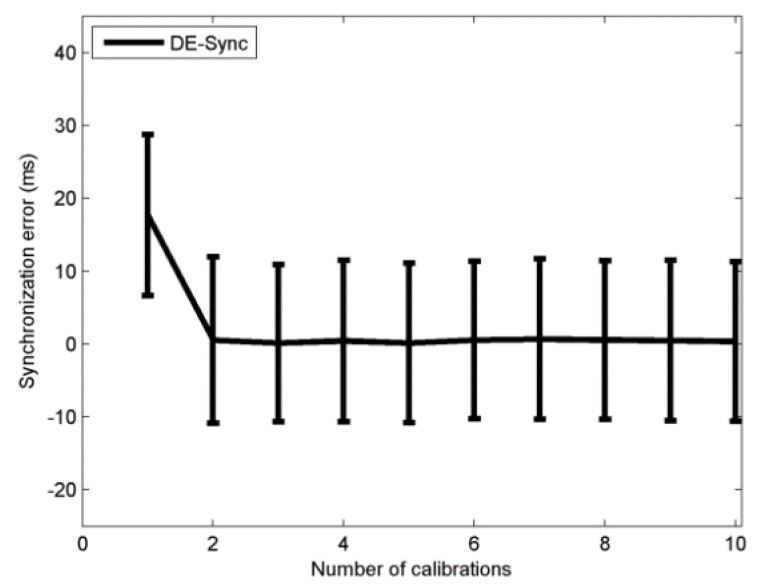
Performance with number of calibrations.

In the calibration process, DE-Sync compensates for the effect of the clock skew in the Doppler scale factor. First, we analyzed the influence of the number of calibrations on the amount of synchronization error. The simulation result is showed in Figure 2, from which we can see that when the number of calibration is 1, the Doppler scale factor is not corrected and the synchronization error is larger. The Doppler scale factor is corrected when the number of calibrations increases, starting from 2. The simulation results show that the synchronization error decreases with an increased number of calibration times, and the performance is enhanced as expected.

#### 4.2.2. Initial Skew

**Figure 3:**
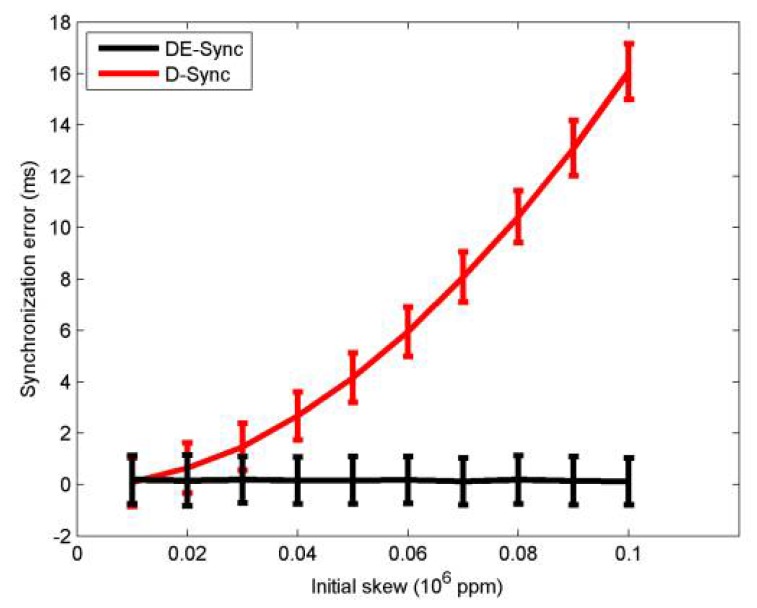
Performance with initial skew.

Due to the effect of the skew during the process of estimating the Doppler scale factor, the DE-Sync performance with its initial skew needs to be analyzed. According to the references [25,26,27,28,32], the inherent skew can be selected with the range from 50 to 10^6^ ppm. However, the inherent skew rarely can reach 10^6^ ppm in practice. Without loss of generality, the simulation is performed with the range of the inherent skew from 10^4^ to 10^5^ ppm. The simulation results are shown in Figure 3, which shows that the synchronization error of the D-Sync deteriorates with an increased skew and DE-Sync has consistent performance across different skews. Notably, DE-Sync significantly outperforms D-Sync when there is a larger skew. In other words, when the initial skew is less than a certain value (10^4^ ppm), the difference of performance between two schemes can be negligible. In the following simulations, we will compare the two schemes using a larger value of the skew (0.05 × 10^6^ ppm).

#### 4.2.3. Response Time

**Figure 4:**
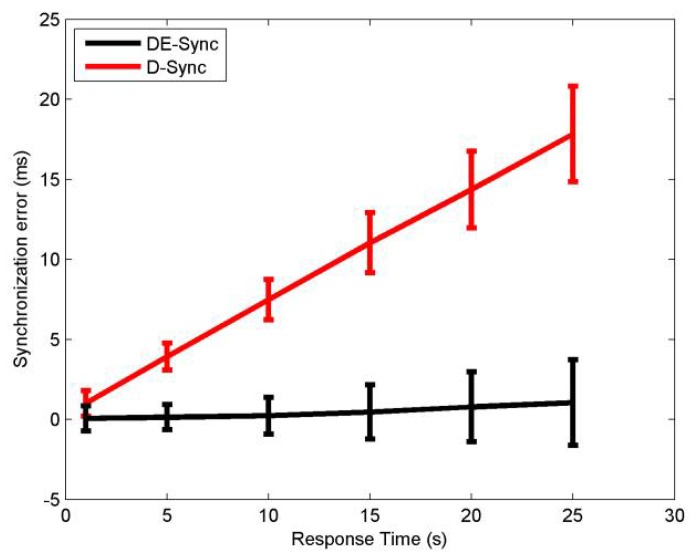
Performance with response time.

Figure 4 shows the mean and standard deviation of synchronization error for DE-Sync and D-Sync when the response time of a node is varied between 1 s and 25 s. As shown in Equation (17), we know that the error in DE-Sync and D-sync increases with the response time because Doppler measurements are available only when a message is exchanged between nodes. However, observing the mean of error, DE-Sync has consistent performance across different response time, because unlike D-Sync, it takes the effect of skew into account during the process of estimating the Doppler scale factor.

#### 4.2.4. Extent of Mobility

**Figure 5:**
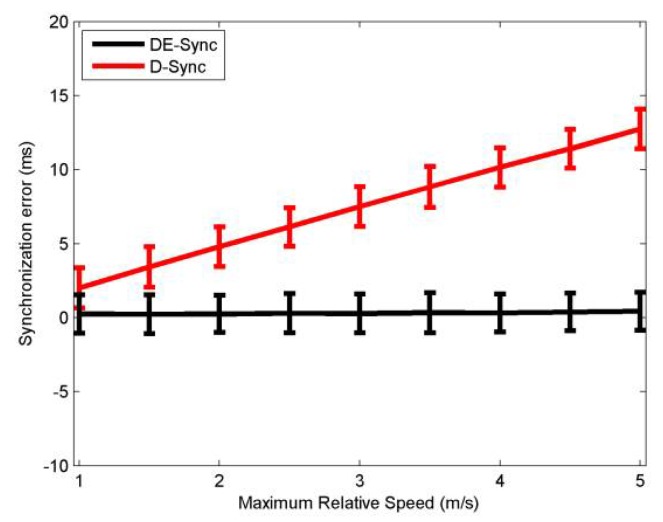
Performance with relative speed.

Figure 5 illustrates the mean and standard deviation of the synchronization error for DE-Sync and D-Sync when the maximum relative speed of a node is varied between 1 m/s and 5 m/s. Based on Equations (3) and (4), without considering the effect of the skew, D-Sync will introduce an estimation error of relative speed *ε_a_*; it can be formulated as:

(19) $$\varepsilon_{a}=0.5\times \left(\alpha +\frac{1}{\alpha }-2\right)\times \left(v_{m}+c\right)$$

Considering Equation (19), if *α =* 1, then *ε_a_ =* 0. However, for unsynchronized nodes, *α* is not equal to 1, which causes *ε_a_* to be larger with the increase of *v_m_*. Further, the mean of the synchronization error of D-Sync deteriorates with increased mobility. As mentioned before, DE-Sync still outperforms D-Sync because it reduces the synchronization error induced by the effect of the skew during the process of estimating the Doppler scale factor. From Figure 5, simulation results are as expected.

**Figure 6:**
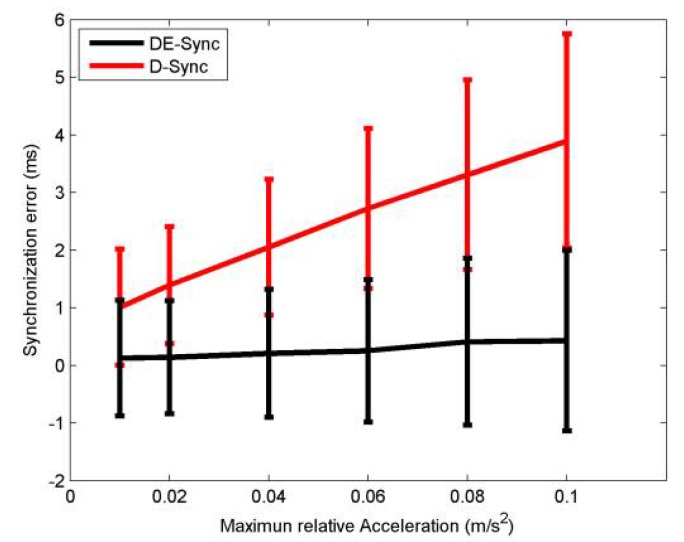
Performance with relative acceleration.

Figure 6 shows the mean and standard deviation of synchronization error for DE-Sync and D-Sync when the maximum relative acceleration of a node is varied between 0.01 m/s^2^ and 0.1 m/s^2^. Based on Equation (18), the accuracy of DE-Sync and D-Sync are affected by the maximum relative acceleration of nodes, in which the synchronization error increases with the increase of the maximum relative acceleration. Due to the fact that the maximum relative speed increases with the increase of maximum relative acceleration, considering Equation (19), we know that the increase of maximum relative speed leads to a greater synchronization error in D-Sync than DE-Sync. From Figure 6, simulation results are consistent with expectations that DE-Sync outperforms D-Sync.

#### 4.2.5. Internal between Request Messages

**Figure 7:**
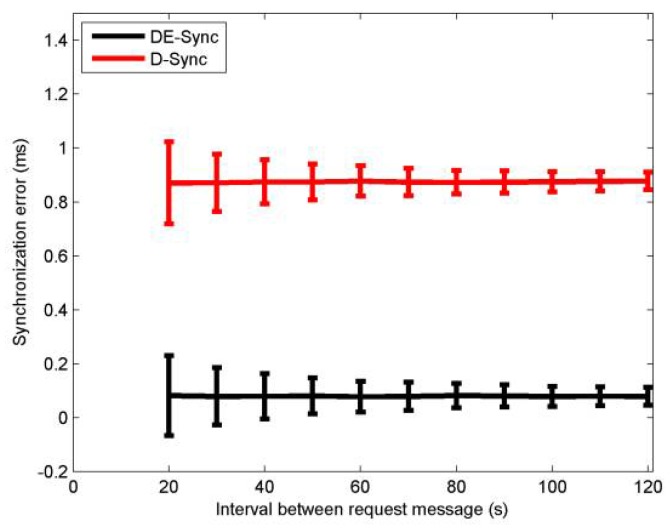
Effect of message interval.

Figure 7 shows the mean and standard deviation of synchronization error for DE-Sync and D-Sync when the time interval between request messages of a node is varied between 20 s and 120 s. Increasing the interval between request messages is equivalent to increasing the dynamic range of linear regression, which allows enough time to elapse before initiating the next message exchange. Performing Equation (13) to enhance the estimation accuracy of the skew and offset, the synchronization error decreases with the increased interval. As described before, DE-Sync has a lower mean of synchronization error than D-Sync. Observing Figure 7, simulation results are in agreement with the expectation that DE-Sync outperforms D-Sync.

#### 4.2.6. Number of Request Messages

**Figure 8:**
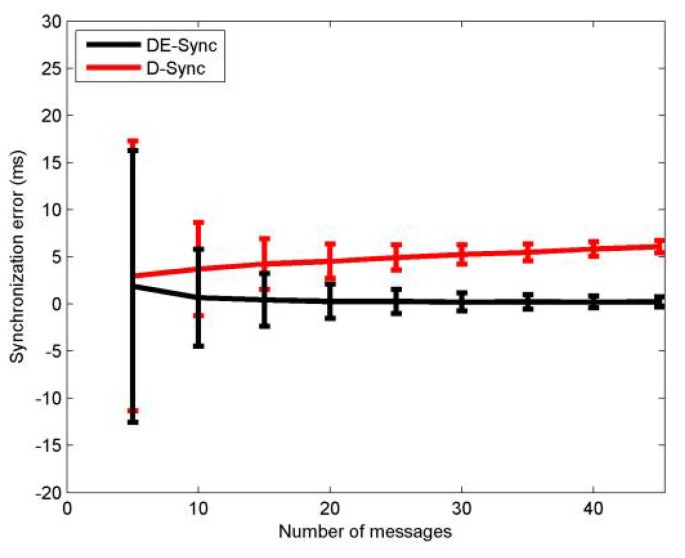
Performance vs number of messages.

Figure 8 shows the mean and standard deviation of synchronization error for DE-Sync and D-Sync when the number of messages of a node is varied between 5 and 45. The standard deviation of synchronization error of DE-Sync and D-Sync decreases with the increase of the number of messages. Meanwhile, we set the acceleration of the node to be non-zero, and with the increase of the number of request messages, the moving speed among nodes also increases. According to the Equation (19), the increase of moving speed leads to the increase of the estimation error of relative speed among nodes in D-sync, so the mean of synchronization error increases with the increase of the number of request messages in D-sync. DE-Sync has a lower mean of synchronization error than D-Sync does. From Figure 8, simulation results are consistent with the expectation that DE-Sync outperforms D-Sync.

#### 4.2.7. Error After Synchronization

**Figure 9:**
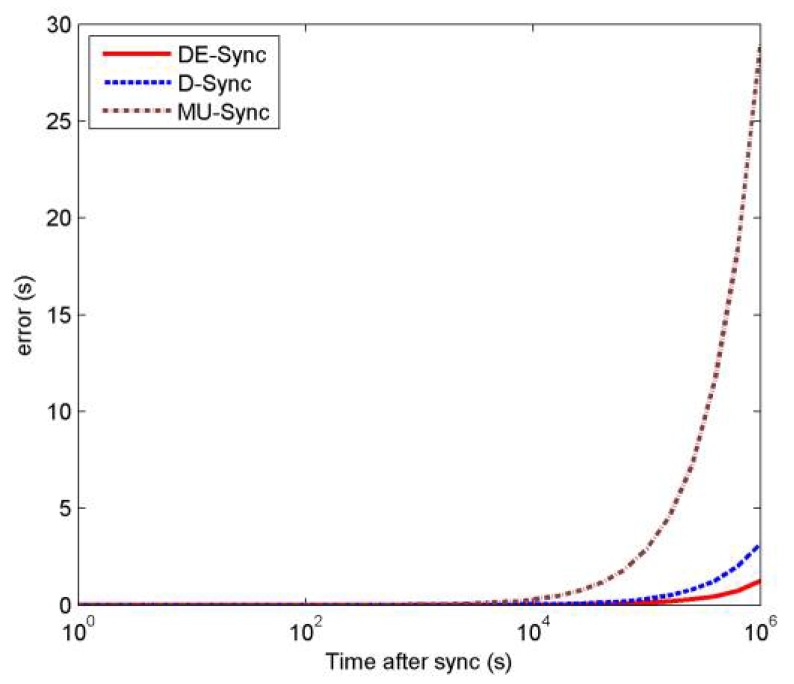
Clock error vs. time after sync.

Figure 9 illustrates how errors grow after time synchronization completes with the three different schemes, namely, MU-Sync, D-Sync and DE-Sync. From this figure, we can see that DE-Sync works much better than MU-Sync and D-Sync, which achieves a more accurate skew, which affects the degree of synchronization error. MU-Sync assumes that the propagation delay of each round of message exchanges is constant, which causes larger errors. D-Sync ignores the effect of the clock skew when estimating the Doppler scale factor. In other words, when the clock skew is larger, the number of synchronization errors rises notably. As described before, DE-Sync corrects the shortcomings of the other schemes, which is the best among all schemes.

#### 4.2.8. Comparison of Energy Efficiency

**Figure 10:**
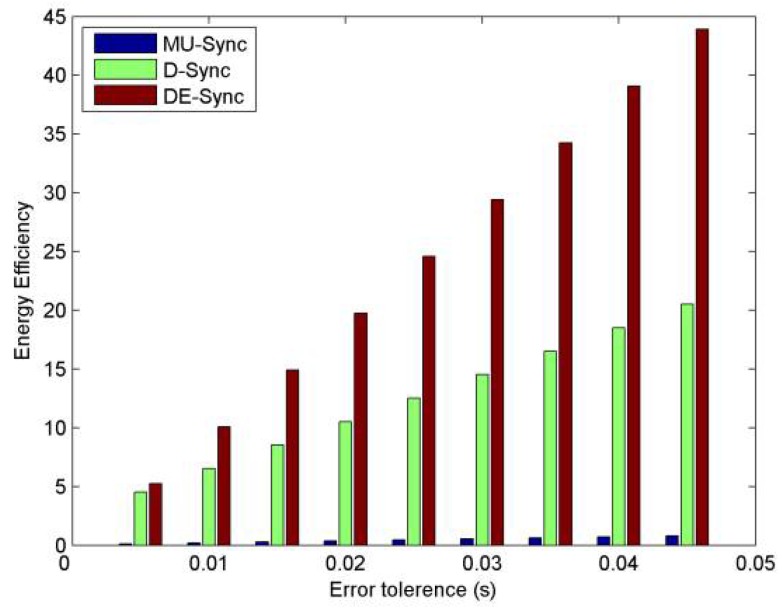
Energy efficiency vs error tolerance.

Figure 10 compares the energy efficiency of the three synchronization approaches. The energy efficiency is directly affected by message-exchange overhead and computational cost. However, the energy cost in computation can actually be negligible compared with the consumption of energy used to send and receive acoustic signals in underwater sensor networks. So ignoring computational cost, in this paper, we use Equation (20) as an evaluation formula of energy efficiency [29].

(20) $$\rho =\frac{\upsilon }{\kappa \mu \gamma }$$

In this equation, *υ* denotes a period of time after time synchronization completes, *υ* is set to 10^6^ s. *μ* and *γ* are the number of messages used in performing a linear regression and the packet size, respectively. *μ* and *γ* are set to 24 messages and 40 Bytes, respectively. *κ* stands for the number of re-synchronization required within a certain duration of *υ* to keep synchronization error below a certain clock error tolerance that is defined as *e*, which is expressed as follows.

(21) $$\kappa =\frac{\upsilon }{\left(\frac{\hat{\alpha }e+\left(\hat{\beta }-\beta \right)}{\alpha -\hat{\alpha }}\right)}$$

Note that, through observing Equation (21), we can know that the estimation accuracy of the skew determines the frequency of re-synchronization, which affects the energy efficiency. In other words, the scheme that has higher accuracy time synchronization will have higher energy efficiency. From this figure, with respect to D-Sync and MU-Sync, DE-Sync has higher energy efficiency, which saves power.

## 5. Conclusions and Future Work

In this paper, we present DE-Sync, a novel synchronization scheme developed for mobile underwater sensor networks, which is a Doppler-enhanced time synchronization. Compared to the existing schemes, DE-Sync does not directly use the Doppler scale factor to calculate the relative speed between nodes. Meanwhile, it conducts linear regression without using the estimates of sound propagation speed, which reduces errors. Furthermore, it compensates for the effect of the skew when using a Doppler scale factor, which improves the accuracy of time synchronization. Meanwhile, when the initial skew reaches a certain value, the performance of DE-Sync will be significantly better in those conditions. Our simulation results show that DE-Sync is a high-precision time synchronization approach with a low message overhead.

Moreover, we will also explore other schemes to enhance the accuracy of synchronization and to reduce the message overhead for mobile underwater sensor networks.

## Figures and Tables

**Table 1:** Parameter Description.

Parameters	Description
*A*	Beacon node
*B*	Unsynchronized node
*α*	Clock skew
*β*	Clock offset
*a_m_*	Doppler scale factor induced by the node mobility
*v_m_*	Average relative speed of nodes
*c*	Sound propagation speed
*a_AB_*	Measured Doppler scaling factor from *A* to *B*
*a_BA_*	Measured Doppler scaling factor from *B* to *A*
*v_AB_*	Measured relative speed from *A* to *B*
*v_BA_*	Measured relative speed from *B* to *A*
*T*_1_	Sending time-stamp of unsynchronized node
*T*_4_	Receiving time-stamp of unsynchronized node
*T*_2_	Receiving time-stamp of beacon
*T*_3_	Sending time-stamp of beacon
*t*_2_, *t*_3_	Reference time at *T*_2_ and *T*_3_
*t*_1_, *t*_4_	Reference time at *T*_1_ and *T*_4_
*T_backoff_*	Response time, that is *T*_3_ − *T*_2_
*τ*_1_	Propagation delay of synchronization request from *B* to *A*
*τ*_2_	Propagation delay of synchronization response from *A* to *B*
Δ*d*	Relative moving distance from *t*_2_ to *t*_4_

**Table 2:** Simulation Parameters.

Parameters	Value
Max distance (*d_max_*)	1000 m
Maximum relative speed (*v_max_*)	5 m/s
Maximum relative acceleration (*A_max_*)	0.1 m/s^2^
Maximum clock skew (*α_max_*)	0.1 × 10^6^ ppm
Clock offset (*β*)	80 ppm
Response time (*T_backoff_*)	1 s
Interval between request messages (*M_interval_*)	3 s
Number of messages (*N*)	25
Clock granularity (*T_gra_*)	1 µs
Reception jitter (*T_jitter_*)	15 µs
